# Kinetics of SARS-CoV-2 specific IgM and IgG responses in COVID-19 patients

**DOI:** 10.1080/22221751.2020.1762515

**Published:** 2020-05-13

**Authors:** Baoqing Sun, Ying Feng, Xiaoneng Mo, Peiyan Zheng, Qian Wang, Pingchao Li, Ping Peng, Xiaoqing Liu, Zhilong Chen, Huimin Huang, Fan Zhang, Wenting Luo, Xuefeng Niu, Peiyu Hu, Longyu Wang, Hui Peng, Zhifeng Huang, Liqiang Feng, Feng Li, Fuchun Zhang, Fang Li, Nanshan Zhong, Ling Chen

**Affiliations:** a State Key Laboratory of Respiratory Diseases, National Clinical Research Center of Respiratory Disease, Guangzhou Institute of Respiratory Health, The First Affiliated Hospital of Guangzhou Medical University, Guangzhou, People’s Republic of China; b Institute of Infectious Diseases, Guangzhou Eighth people’s Hospital, Guangzhou Medical University, Guangzhou, People’s Republic of China; c Guangzhou Regenerative Medicine and Health-Guangdong Laboratory (GRMH-GDL), Guangzhou Institutes of Biomedicine and Health, Chinese Academy of Sciences, Guangzhou, People’s Republic of China

**Keywords:** COVID-19, SARS-CoV-2, IgM, IgG, C-reactive protein

## Abstract

The emerging COVID-19 caused by SARS-CoV-2 infection poses severe challenges to global public health. Serum antibody testing is becoming one of the critical methods for the diagnosis of COVID-19 patients. We investigated IgM and IgG responses against SARS-CoV-2 nucleocapsid (N) and spike (S) protein after symptom onset in the intensive care unit (ICU) and non-ICU patients. 130 blood samples from 38 COVID-19 patients were collected. The levels of IgM and IgG specific to N and S protein were detected by ELISA. A series of blood samples were collected along the disease course from the same patient, including 11 ICU patients and 27 non-ICU patients for longitudinal analysis. N and S specific IgM and IgG (N-IgM, N-IgG, S-IgM, S-IgG) in non-ICU patients increased after symptom onset. N-IgM and S-IgM in some non-ICU patients reached a peak in the second week, while N-IgG and S-IgG continued to increase in the third week. The combined detection of N and S specific IgM and IgG could identify up to 75% of SARS-CoV-2 infected patients in the first week. S-IgG was significantly higher in non-ICU patients than in ICU patients in the third week. In contrast, N-IgG was significantly higher in ICU patients than in non-ICU patients. The increase of S-IgG positively correlated with the decrease of C-reactive protein (CRP) in non-ICU patients. N and S specific IgM and IgG increased gradually after symptom onset and can be used for detection of SARS-CoV-2 infection. Analysis of the dynamics of S-IgG may help to predict prognosis.

## Introduction

Since December 2019, cases of unexplained pneumonia have occurred in Wuhan City, Hubei Province, subsequent virus isolation and whole-genome sequencing (accession#: MN908947) confirmed that it is an acute respiratory infection caused by new severe acute respiratory syndrome coronavirus 2 (SARS-CoV-2) [1,2]. Coronaviruses are enveloped, non-segmented, single-positive-stranded RNA viruses with round or oval particles and a diameter of 50–200 nm. Coronavirus subfamily is divided into four genera: *α*, *β*, *γ* and *δ* according to serotype and genomic characteristics. The SARS-CoV-2 belongs to the genus *β* which has been confirmed to be highly infectious by research. As of April 20, 2020, SARS-CoV-2 has caused more than 2446840 laboratory-confirmed human infections, including 170993 deaths, posing a serious threat to human health (https://www.who.int/emergencies/diseases/novel-coronavirus-2019/situation-reports).

The four major structural proteins of coronavirus are the spike surface glycoprotein (S), small envelope protein (E), matrix protein (M), and nucleocapsid protein (N). The spike protein (S) of coronavirus is a type I transmembrane glycoprotein and mediates the entrance to human respiratory epithelial cells by interacting with cell surface receptor angiotensin-converting enzyme 2 (ACE2) [3], the S protein contains distinct functional domains near the amino (S1) and carboxy (S2) termini, the peripheral S1 portion can independently bind cellular receptors while the integral membrane S2 portion is required to mediate fusion of viral and cellular membranes [4–6]. The nucleocapsid protein (N) forms complexes with genomic RNA, interacts with the viral membrane protein during virion assembly and plays a critical role in enhancing the efficiency of virus transcription and assembly [7–9].

The SARS-CoV-2 has human-to-human transmission characteristics and a high fatality in critically ill patients. Compared with non-ICU patients, ICU patients had higher plasma levels of IL2, IL6, IL7, IL10, GSCF, IP10, MCP1, MIP1A, TNF*α*, lactate dehydrogenase (LDH), ferritin and D-dimer. The number of lymphocytes was significantly reduced and C-reactive protein (CRP) was significantly increased in severe cases [10–13]. During the submission of this paper, several publications have also reported the analysis of antibody responses to N protein, S protein, and receptor-binding domain (RBD) on S protein in COVID-19 patients [14–18]. However, the seropositive rate of both IgM and IgG responses within one week after onset and in the context of both N protein and S protein has not been clarified. The kinetics of antibody responses in critical cases or ICU patients has not been reported, and no studies have suggested whether antibody response is associated with disease prognosis. Here, we systemically investigated the kinetics of IgG and IgM responses to both N and S proteins in the first 4 weeks after the symptom onset in ICU and non-ICU patients. Our study can help to facilitate serologically based diagnosis and prediction of disease prognosis.

## Materials and methods

### Source of serum samples

One hundred thirty blood samples from 38 patients were collected between 3 and 28 days after symptom onset. Blood samples from non-ICU patients with confirmed SARS-CoV-2 infection were collected from 27 non-ICU patients from the Guangzhou Eighth People’s Hospital. Blood samples from 11 ICU patients were collected from the First Affiliated Hospital of Guangzhou Medical University. 16 negative serum samples were collected from healthy volunteers. The serum samples were separated after centrifugation at 3,000 rpm for 10 min, and then inactivated at 56°C for 1.5 h.

### Enzyme-linked immunosorbent assay (ELISA)

SARS-CoV-2 N protein and S protein-specific binding antibodies were analyzed by ELISA as described previously [19]. N protein (residue 1–419) was produced from Baculovirus-Insect Cells (Cat. # 40588-V08B, Sino biological, Beijing, China). S protein (residue 16–685) was produced from HEK293 Cells (Cat. #40591-V08H, Sino biological, Beijing, China). The specificity of SARS-CoV-2 N and S proteins were verified by Western blot analysis using serum samples from convalescent COVID-19 patients and from other respiratory pathogens (influenza virus, adenovirus, and human coronaviruses OC43 and HKU1) infected patients (Supplementary Fig. 1). Microtiter plates were coated with 50 ng/well of target protein overnight at 4 °C. Plates were then blocked for 2 h at 37°C using 200 μL of 5% non-fat milk in 1 x phosphate buffered saline (PBS). Serum samples were then diluted 1:50 in 1X PBS and 100 μL of each sample was applied to coated ELISA plate and incubated for 2 h at 37 °C. Plates were then washed and incubated with HRP-labeled anti-human IgM and IgG (Sigma Aldrich, MI, USA), diluted to 1:2000 in 5% non-fat milk in 1 x PBS. After incubation for another 1 h at room temperature, the plates were washed and developed with TMB/E substrate (Merck Millipore, MA, USA). Finally, the reaction was stopped with 1M H_2_SO_4_, and the OD450 nm values were read. Negative serum control was run each time the assay was performed. The cut-off value for seropositivity samples was set as the mean value at optical density 450 (at a 1:50 dilution) for the 16 negative serum samples plus 3 standard deviations (SDs).

### Ethics approval

This study was approved by the First Affiliated Hospital of Guangzhou Medical University and Guangzhou Eighth people’s Hospital. Written informed consent was waived for in the light of this emerging infectious disease of high clinical relevance. All healthy control subjects signed written informed consent before the collection of peripheral blood.

### Statistical analysis

Statistical analyses and graphical presentations were conducted with GraphPad Prism version 7.0 (GraphPad Software, Inc., CA, USA). We compared categorical variables of basic clinical characteristics of ICU and non-ICU patients using Fisher’s exact test. Differences of antibody responses between ICU and non-ICU patient groups were determined by Student’s t test. Throughout the text, figures, and legends, the following terminology is used to show statistical significance: *, *P* < 0.05; **, *P* < 0.01; and ***, *P* < 0.001.

## Results

### N and S specific IgM and IgG were detectable in 75% non-ICU patients in the first week after symptom onset

The basic information and clinical symptoms of 27 non-ICU patients (14 male and 13 female) and 11 ICU patients (10 male and 1 female) are summarized (Table 1). The non-ICU patients had a median age 44.0 (interquartile range, IQR: 32.0–56.0), SARS-COV-2 nucleic acid positive days of 13.0 (IQR: 12.0–16.3), and median hospitalization days of 19.0 (IQR: 14.3–22.5). The ICU patients had a median age 58.0 (IQR: 49.0–69.5), SARS-CoV-2 nucleic acid positive days of 31.0 (IQR: 22.5–32.0) or longer, and median hospitalization days of 31.0 (IQR: 30.0–33.5) or longer. The levels of N-IgM, N-IgG, S-IgM, S-IgG were measured by ELISA. Serum samples from 16 healthy people were used as negative controls. The cut-off value for seropositivity samples was set as the mean value at optical density 450 (at a 1:50 dilution) for the 16 negative serum samples plus 3 standard deviations (SDs), which were 0.394, 0.291, 0.284 and 0.170 for N-IgM, N-IgG, S-IgM and S-IgG, respectively.

**Table 1.** Basic information of COVID-19 patients.

	Group A: non-ICU patients (*n* = 27)	Group B: ICU patients (*n* = 11)	*P* value
Median ages (IQR)	44.0 (32.0–56.0)	58.0 (49.0–69.5)	0.05
Gender			
Female	13(48%)	1(9%)	0.03
Male	14(52%)	10(91%)	..
Median days of admission after symptom onset (IQR)			
	4(3.75–7)	5(2–10.5)	0.35
Median hospital Stay days (IQR)			
	19.0 (14.3–22.5)	31.0 (30.0–33.5)	<0.001
Median days of SARS-COV-2 Nucleic Acid negative after symptom onset (IQR)			
	13.0 (12.0–16.3)	31.0 (22.5–32.0)	<0.001
Presenting symptoms			
Fever	26(96%)	10(91%)	0.5
Cough	22(81%)	11(100%)	0.29
Shortness of breath	5(19%)	8(73%)	0.003
Anorexia	8(30%)	10(91%)	<0.001
Underlying medical disorders			
None	14(52%)	5(45%)	>0.99
Hypertension	5(19%)	4(36%)	0.4
Chronic pulmonary disease	0(0%)	2(18%)	0.08
Coronary heart disease	1(4%)	0(0%)	>0.99
Chronic gastritis	2(7%)	0(0%)	>0.99
Liver cyst	1(4%)	0(0%)	>0.99
Tuberculosis	1(4%)	0(0%)	>0.99
Hyperlipidemia	1(4%)	0(0%)	>0.99
Fatty liver	1(4%)	0(0%)	>0.99
Thalassemia	1(4%)	0(0%)	>0.99
Colon cancer	1(4%)	0(0%)	>0.99
Diabetes	0(0%)	5(45%)	<0.001

IQR: Interquartile range.

**Figure 1.**
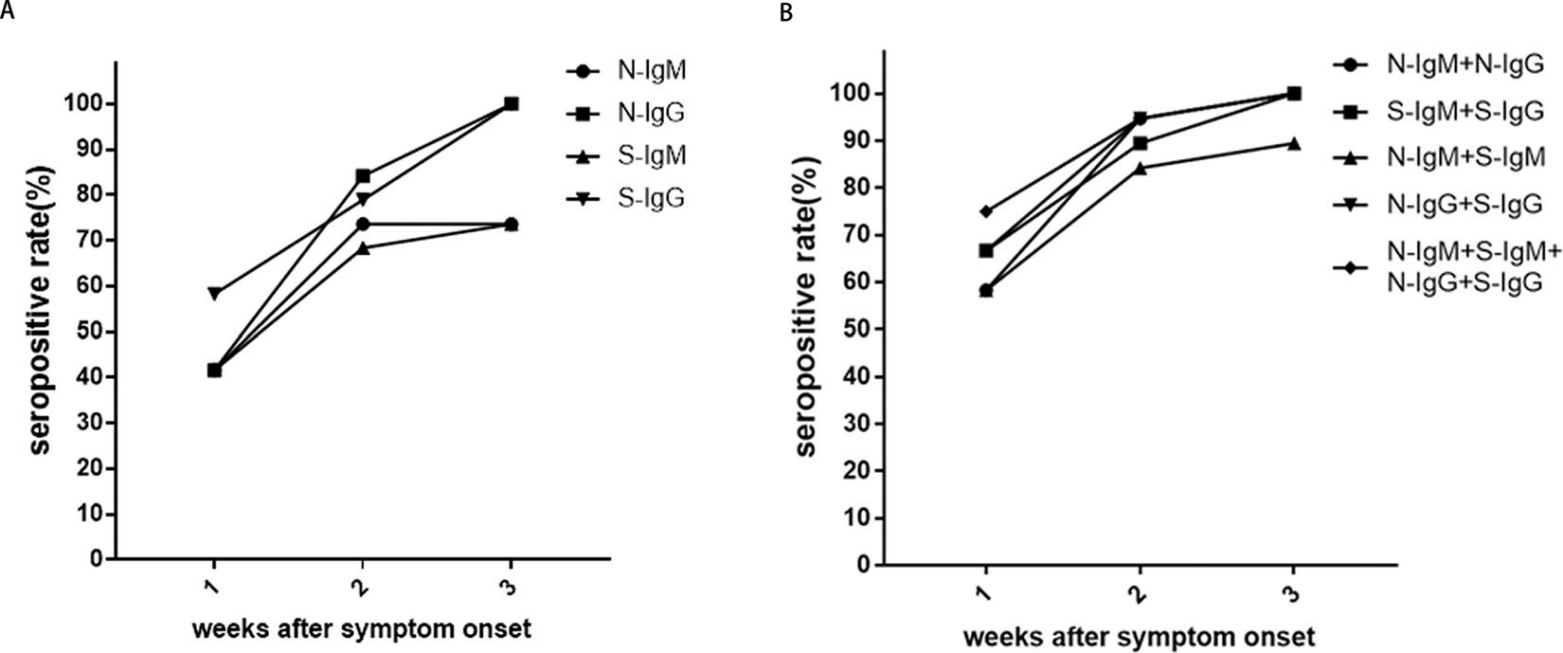
The seropositive rates of N and S specific IgM and IgG antibody responses in non-ICU patients after symptom onset. A. The changes in seropositive rates of N-IgM, N-IgG, S-IgM and S-IgG in 27 non-ICU patients. B. The changes in seropositive rates of N-IgM + N-IgG, S-IgM + S-IgG, N-IgM + S-IgM, N-IgG + S-IgG, N-IgM + S-IgM + N-IgG + S-IgG in 27 non-ICU patients.

The results showed that within one week after the symptom onset, the seropositive rates of N-IgM, N-IgG and S-IgM in non-ICU patients were 41.7%, and the seropositive rate of S-IgG was 58.3%. The seropositive rate of N-IgM + N-IgG, N-IgM + S-IgM were 58.3%, while the seropositive rate of S-IgM + S-IgG, N-IgG + S-IgG reached 66.7%. The seropositive rate of N-IgM + S-IgM + N-IgG + S-IgG reached 75.0% (Table 2). This result indicated that the combined detection of N and S specific IgM and IgG can be useful for early detection of SARS-CoV-2 infection. In the second weeks after symptom onset, the seropositive rates were 73.7% for N-IgM, 68.4% for S-IgM, 84.2% for N-IgG, and 78.9% for S-IgG. The seropositive rate of N-IgM + S-IgM was 84.2%, while the seropositive rate of N-IgM + N-IgG, N-IgG + S-IgG reached 94.7%. In the third weeks after symptom onset, the seropositive rates of either N-IgM or S-IgM maintained at 73.7%, while the seropositive rates of N-IgG and S-IgG reached 100% (Table 2). This result showed that the seropositive rates of N-IgM, S-IgM, N-IgG, and S-IgG responses increased with disease course in non-ICU patients (Figure 1A, B).

**Table 2.** Seropositive rate (%).

Weeks	N-IgM	N-IgG	S-IgM	S-IgG	N-IgM + N-IgG	S-IgM + S-IgG	N-IgM + S-IgM	N-IgG + S-IgG	N-IgM + S-IgM + N-IgG + S-IgG
1	41.7	41.7	41.7	58.3	58.3	66.7	58.3	66.7	75.0
2	73.7	84.2	68.4	78.9	94.7	89.5	84.2	94.7	94.7
3	73.7	100.0	73.7	100.0	100.0	100.0	89.5	100.0	100.0

N-IgM: N protein specific IgM; N-IgG: N protein specific IgG; S-IgM: S protein specific IgM; S-IgG: S protein specific IgG.

### Kinetics of N-IgM, S-IgM, N-IgG and S-IgG had different patterns in non-ICU and ICU patients

**Figure 2.**
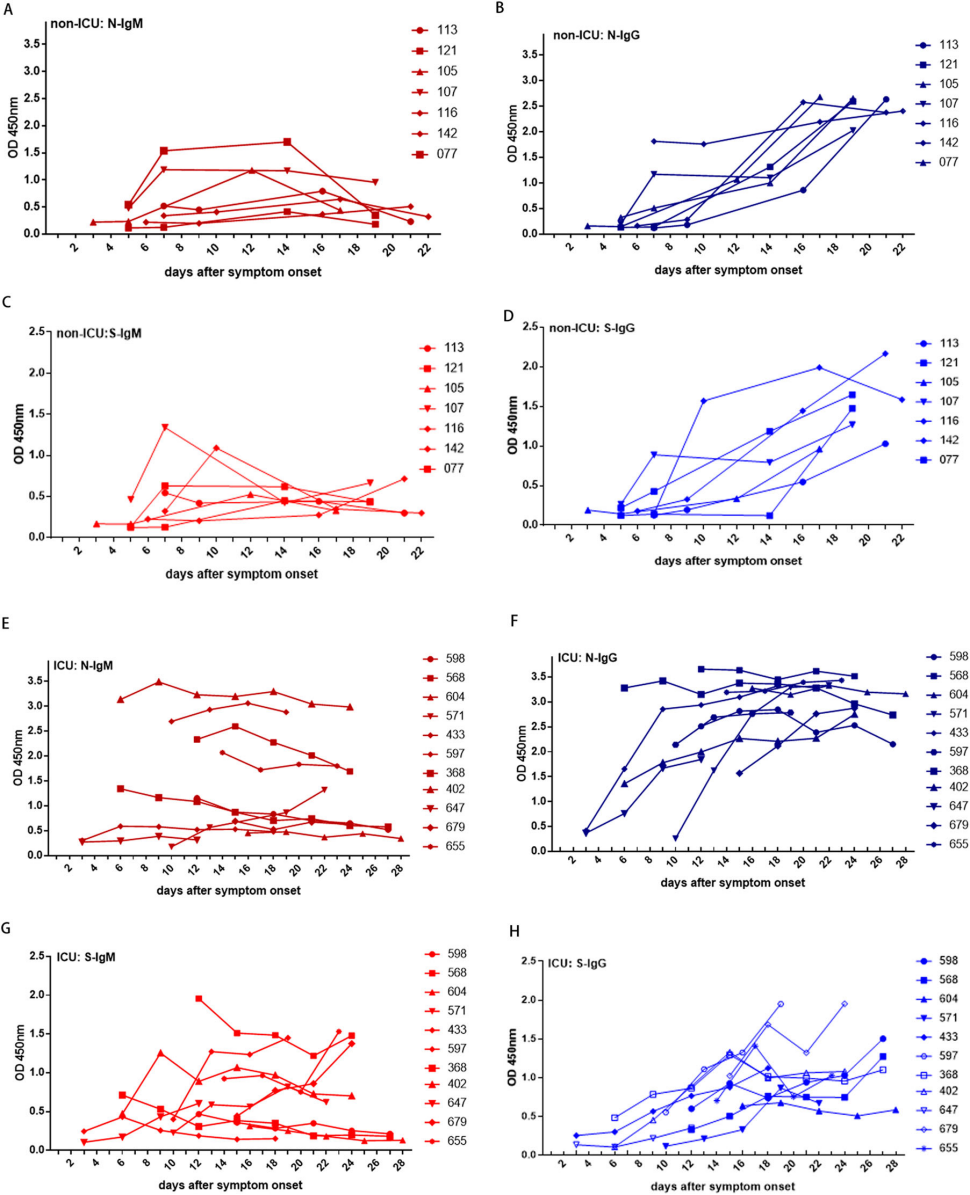
Kinetics of N and S specific IgM and IgG responses in non-ICU patients and ICU patients. (A) N-IgM, (B) N-IgG, (C) S-IgM, (D) S-IgG responses in 7 non-ICU patients; (E) N-IgM, (F) N-IgG, (G) S-IgM, (H) S-IgG antibodies response in 11 ICU patients.

In most non-ICU patients, N-IgM and S-IgM reached a peak in the second week after symptom onset (Figure 2A, 2C, Supplementary Fig.2). Longitudinal analysis showed a decline for N-IgM and S-IgM in the third week after the onset in some non-ICU patients (Figure 2A, 2C, Supplementary Fig.2). In the first week after onset, the levels of N-IgM and N-IgG, S-IgM and S-IgG were similar. N-IgG had a parallel or similar dynamic pattern as N-IgM in the first two weeks for the same patient. However, in some patients, N-IgM showed plateau or declined in the third week while N-IgG continued to increase. The level of N-IgG surpassed N-IgM in the second and third week after onset (Table 3). S-IgG also had a parallel or similar dynamic pattern as S-IgM for the same person in the first two weeks for non-ICU patients. In the third week, the level of S-IgG continued to increase and surpassed the level of S-IgM in the same patient (Figure 2A, B, C, D, Supplementary Fig.2), suggesting that there was an IgM to IgG class-switch from in most non-ICU patients.

**Table 3.** SARS-COV-2 N and S specific IgM and IgG responses (OD450nm: mean + SD).

Weeks after onset	Patients	N		*P* value	S		*P* value	The ratio of N-IgG/S-IgG
		IgM	IgG		IgM	IgG		
Week1	non-ICU (*n* = 14)	0.44 + 0.41	0.51 + 0.53	0.358	0.36 + 0.32	0.24 + 0.20	0.132	2.56 + 3.09
	ICU (*n* = 6)	0.99 + 1.03	1.31 + 1.0	0.316	0.36 + 0.21	0.23 + 0.14	0.145	6.04 + 3.50
	*P* value	0.061	0.020		0.500	0.481		0.025
Week2	non-ICU (*n* = 19)	0.51 + 0.28	1.0 + 0.69	0.004	0.42 + 0.27	0.58 + 0.49	0.112	2.87 + 3.45
	ICU (*n* = 15)	1.51 + 1.12	2.38 + 0.86	0.014	0.69 + 0.48	0.57 + 0.28	0.216	5.98 + 5.10
	*P* value	0.000	0.000		0.026	0.467		0.024
Week3	non-ICU (*n* = 20)	0.6 + 0.38	1.93 + 0.73	0.001	0.64 + 0.52	1.25 + 0.62	0.001	2.11 + 1.67
	ICU (*n* = 25)	1.50 + 1.01	2.92 + 0.52	<0.001	0.70 + 0.43	1.01 + 0.36	0.025	3.38 + 1.67
	*P* value	<0.001	<0.001		0.335	0.028		0.011

ICU: intensive care unit. N-IgG: N protein specific IgG; S-IgG: S protein specific IgG.

In ICU patients, the dynamic patterns of N and S IgM and IgG were more chaotic. N-IgM in 63.6% of ICU patients appeared to remain at low and static levels, while in 36.3% of ICU patients N-IgM had the high but static level for at least 4 weeks (Figure 2E). N-IgG levels in all ICU patients reached high levels (OD450 > 2.0) within 2 weeks after symptom onset (Figure 2F). In 81.8% of ICU patients, N-IgG exceeded N-IgM levels in the same patient by 2 weeks after symptom onset (Figure 2F, Supplementary Fig. 3, A-E, G, I, J, K). N-IgG was significantly higher than N-IgM in the second and third week after onset in ICU patients (Table 3, Supplementary Fig. 3). S-IgM had either poor responses or maintained a static but high level in ICU patients (Figure 2G, Table 3, Supplementary Fig. 3). S-IgG appeared to increase slowly as compared to the increase of N-IgG (Figure 2H, Table 3, Supplementary Fig. 3). In the third week after onset, S-IgG was higher than S-IgM in most ICU patients (Table 3, Supplementary Fig. 3).

**Figure 3.**
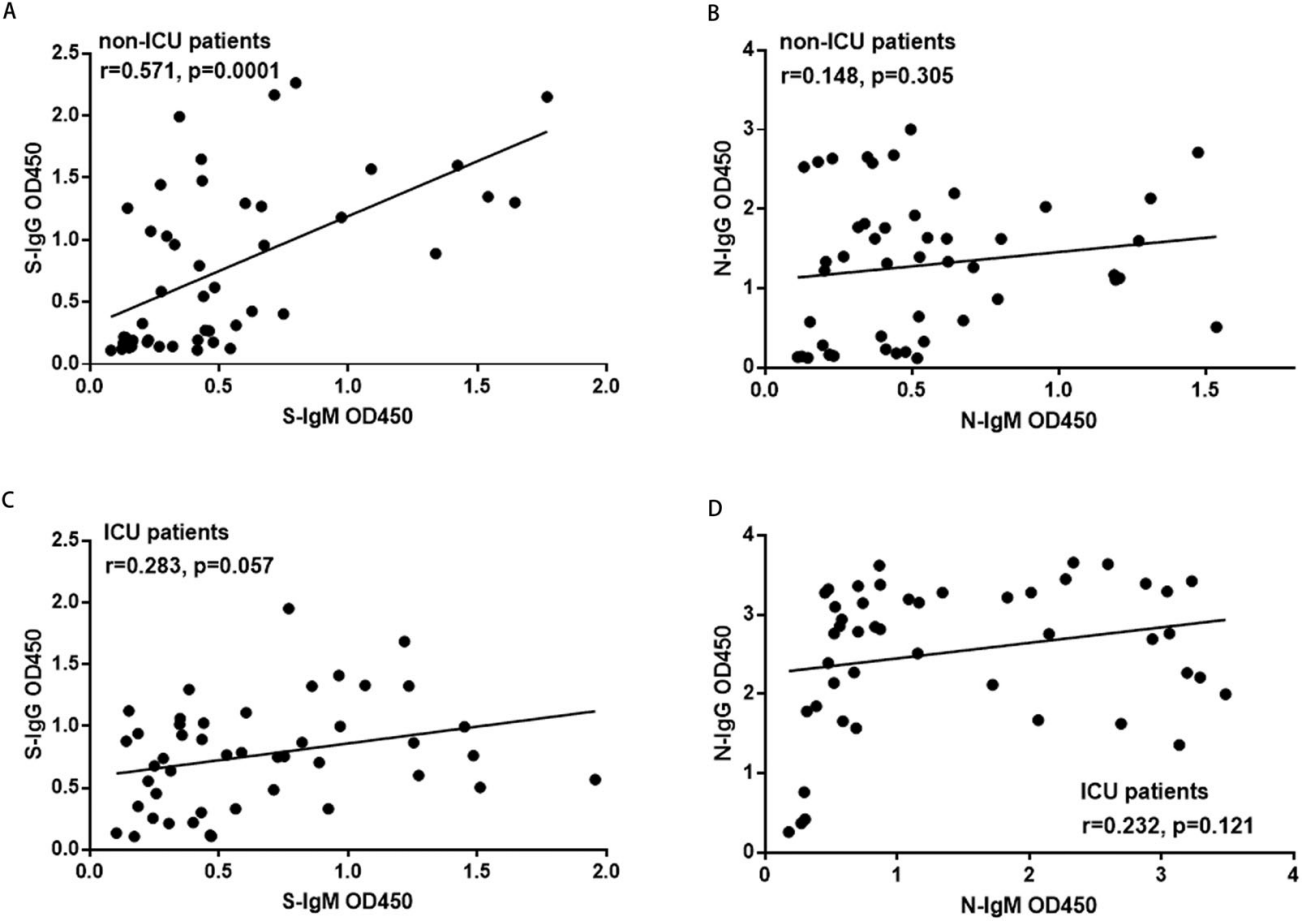
The correlation between N and S specific IgM and IgG responses in non-ICU patients and ICU patients. A. The correlation between S-IgG and S-IgM in non-ICU patients; B. The correlation between N-IgG and N-IgM in non-ICU patients; C. The correlation between S-IgG and S-IgM in ICU patients; D. The correlation between N-IgG and N-IgM in ICU patients. The Pearson correlation coefficient was used to measure the strength of the correlation between IgM and IgG antibodies. The correlation coefficient was calculated using Student’s t-test, a *P*-value < 0.05 was considered statistically significant. *, *P* < 0.05; **, *P* < 0.01; ***, *P* < 0.001.

The correlation between the corresponding S-IgM, S-IgG, N-IgM, and N-IgG levels in each patient were analyzed (Figure 3). In non-ICU patients, there was a strong correlation between the S-IgG with S-IgM levels, whereas there was no correlation between N-IgM with N-IgG levels. In ICU patients, there were no correlations either between S-IgG with S-IgM or between N-IgG with N-IgM levels. The S-IgG levels showed a higher correlation with N-IgG levels in non-ICU patients (correlation coefficient *r* = 0.692, *P* = 0.0001) than in ICU patients (correlation coefficient *r* = 0.377, *P* = 0.01) (Supplementary Fig.4B, D).

### The increase of S-IgG positively correlated with the decrease of CRP in non-ICU patients

**Figure 4.**
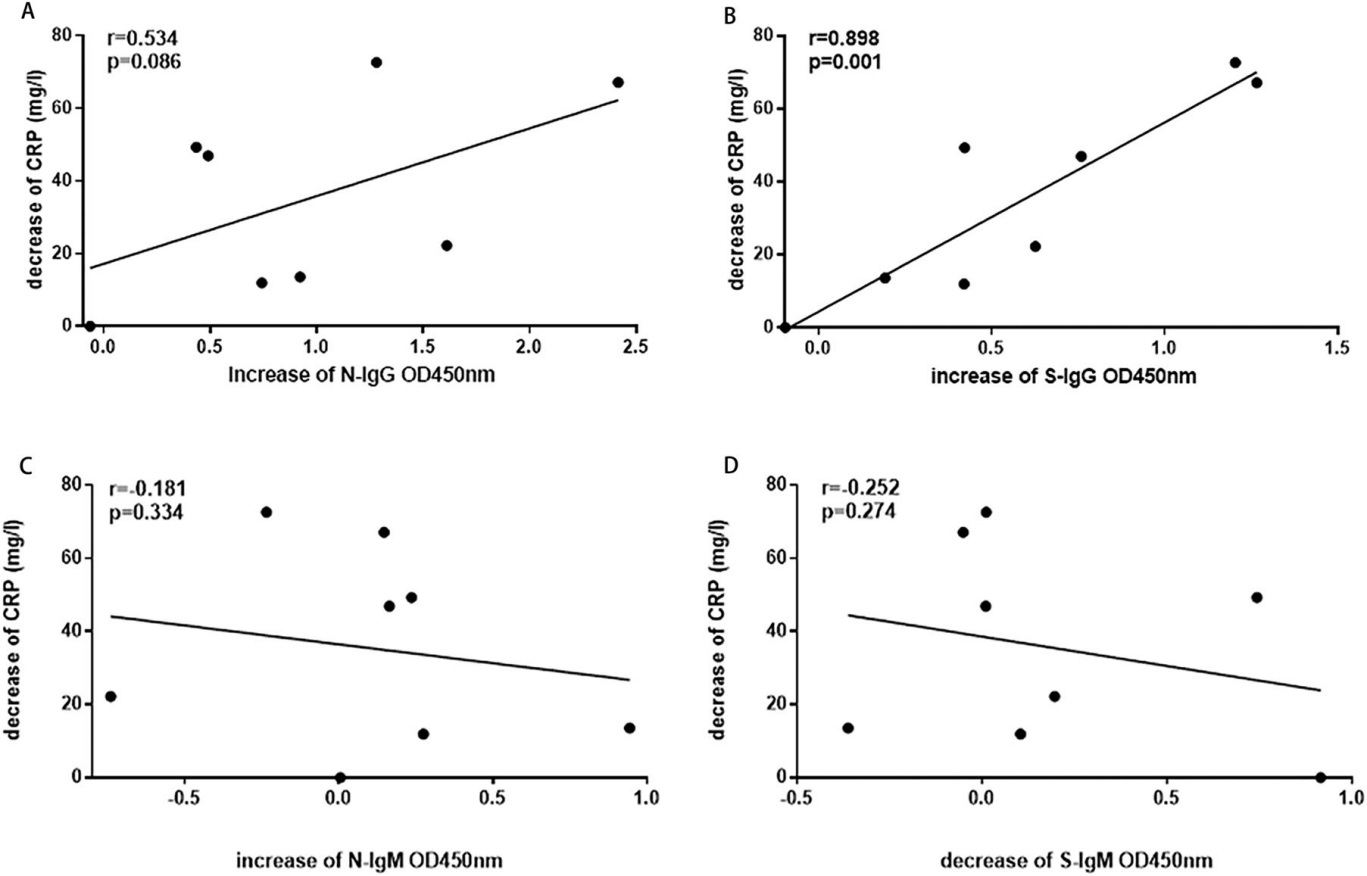
The correlation between N and S specific IgM and IgG responses with CRP in non-ICU patients. A. The correlation between N-IgG and the reduction of CRP; B. The correlation between S-IgG and the reduction of CRP; C. The correlation between N-IgM and CRP; D. The correlation between S-IgM and CRP. The Pearson correlation coefficient was used to measure the strength of the correlation between CPR and IgM or IgG antibodies.

C-reactive protein (CRP) is an acute protein that rises sharply in the plasma when the body is infected or the tissue is damaged. It is a non-specific marker of inflammation and directly participates in the host defence against infection. The levels of N and S specific IgM and IgG were evaluated for correlations with CRP levels in non-ICU patients. As the disease progressed, the increase of S-IgG positively correlated with the decrease of CRP in non-ICU patients (Figure 4B), the correlation coefficients r were 0.9 (*P* = 0.001). However, the changes of N-IgG showed no correlation with the changes of CRP in non-ICU patients (Figure 4A). The changes of N-IgM, and S-IgM also showed no significant correlations with CRP in non-ICU patients (Figure 4C, D).

### S-IgG was significantly lower in ICU patients than in non-ICU patients in the third weeks after symptom onset

**Figure 5.**
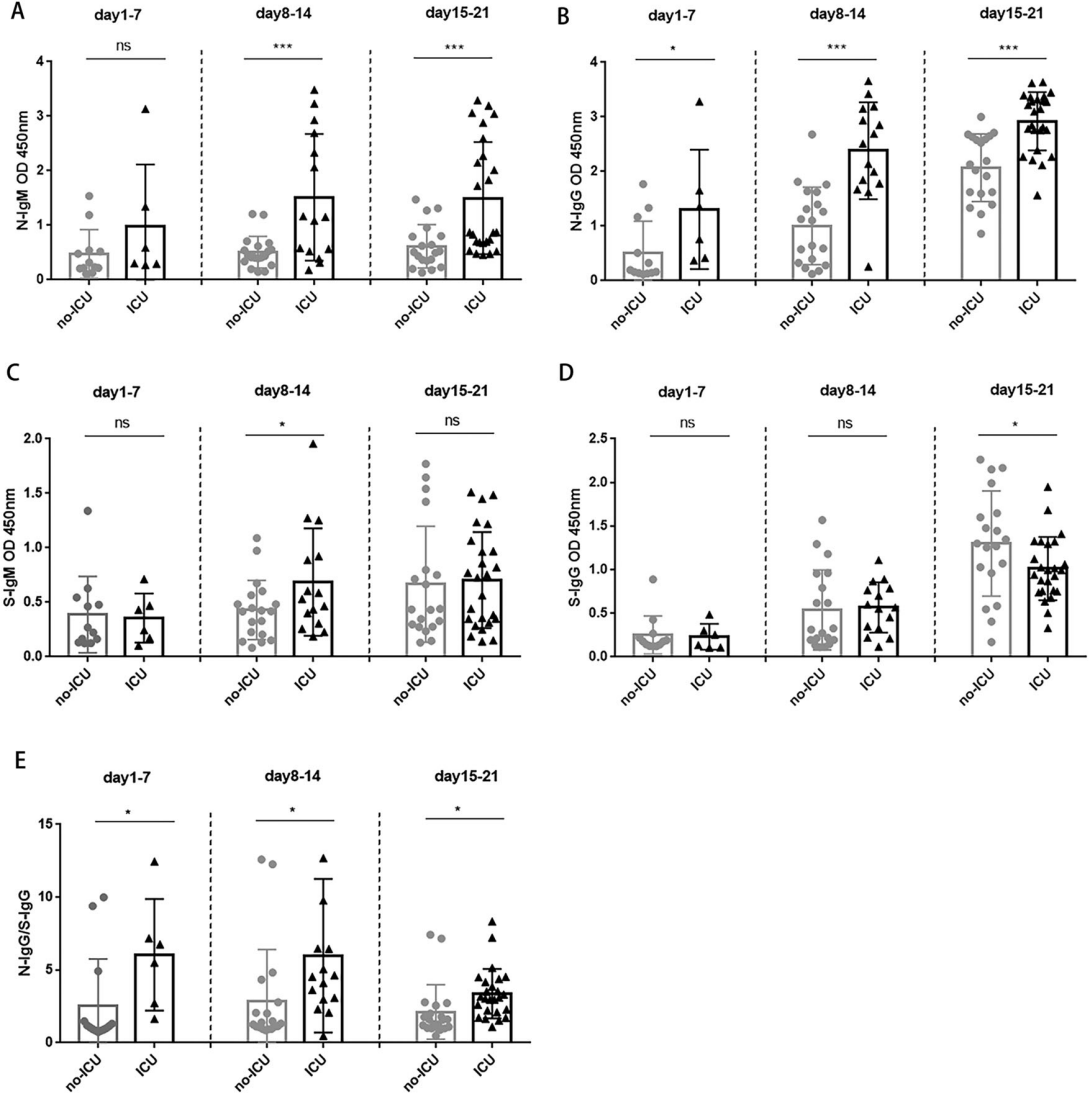
The N and S specific IgM and IgG responses in non-ICU patients and ICU patients. A. Comparison of N-IgM responses between non-ICU and ICU patients; B. Comparison of N-IgG responses between non-ICU and ICU patients; C. Comparison of S-IgM responses between non-ICU and ICU patients; D. Comparison of S-IgG responses between non-ICU and ICU patients. E. Comparison of N-IgG/S-IgG ratio between non-ICU and ICU patients. Correlation coefficient was calculated using Student’s t test, a *P*-value < 0.05 was considered statistically significant. *, *P* < 0.05; **, *P* < 0.01; ***, *P* < 0.001.

In the second and third week after symptom onset, N-IgM was significantly higher in ICU patients than in non-ICU patients (Figure 5A, Table 3, *P *< 0.001). N-IgG was significantly higher in ICU patients than in non-ICU patients after onset (Figure 5B, Table 3, *P *< 0.05). S-IgM was significantly higher in ICU patients than non-ICU patients only in the second weeks after symptom onset (Figure 5C, Table 3, *P *< 0.05). In contrast, S-IgG was significantly lower in ICU patients than in non-ICU patients in the third weeks after symptom onset (Figure 5D, Table 3 *P *< 0.05). Moreover, N-IgG/S-IgG ratio was significantly higher in ICU patients that non-ICU patients throughout the disease course (Figure 5E, *P *< 0.05). ICU patients tended to produce more N-IgM and N-IgG than non-ICU patients. Non-ICU patients tended to have faster and higher IgM to IgG class switch than ICU patients (Table 3, Supplementary Fig.2, Supplementary Fig.3). This result suggested that the class switch of S-IgM to S-IgG is vital for clearing the viruses and can be used as a prognosis indicator to predict the outcome of COVID-19 disease.

## Discussion

This study investigated the kinetics of N and S specific IgM and IgG responses in COVID-19 patients after symptom onset. A total of 130 blood samples from 38 COVID-19 patients were analyzed. Our study showed that the seropositive rates of N-IgM, N-IgG, S-IgM and S-IgG antibody responses in non-ICU patients gradually increased within 1–3 weeks after the onset. N-IgM and S-IgM reached a peak in the second week, while N-IgG and S-IgG antibodies continued to increase in the third week. Joint detection of N-IgM, N-IgG, S-IgM, and S-IgG antibodies, could detect up to 75% of infections in the first week. Joint detection of N-IgM + N-IgG, or N-IgG + S-IgG could detect up to 94.7% of infections in the second week. In the third weeks after symptom onset, seropositive rates for N-IgG and S-IgG reached 100%. In contrast, seropositive rates for N-IgM and S-IgM remained the same as some patients started to decline as the result of IgM to IgG isotype switch, which may help to generate more effective antibodies that can inhibit virus infection.

The effective method to control the spread of the virus is the early diagnosis and early isolation of patients. However, the incubation period of the SARS-CoV-2 and the limitation of Q-PCR for nucleic acid detection affect the positive rate of early diagnosis. It has been reported that the serum antibody ELISA and Q-PCR combined detection may increase the positive rate for the early diagnosis of COVID-19 infection [20–23]. During the preparation of this manuscript, a paper reported antibody detection in 23 COVID-19 patients. The seropositivity rates were 94% for anti-NP IgG, 88% for anti-NP IgM, 100% for anti-RBD IgG, and 94% for anti-RBD IgM [21]. However, the blood samples were collected at 14 days or later after symptom onset, but not in the first week as our study, which can be more useful for diagnostic purpose. It is also important to note that RBD only represents a small part of S protein (237 amino acids in RBD as compared to1273 amino acids in S protein). Therefore, the anti-RBD IgM and anti-RBD IgG response may not represent the antibody response to S protein. Based on our study, we proposed that the combined detection of both N and S specific IgM and IgG may improve the serological detection rate SARS-CoV-2 infection in the early stage. While the decline of IgM/IgG ratio may help to identify the post-infected people, although it is still too early to know when IgM will wane over time. There may be a concern that potential cross-reactivity of SARS-CoV-2 N proteins with other human coronaviruses, which may affect the seropositive rate of SARS-COV-2. An earlier analysis showed that there were no cross-reactivity of SARS-CoV-2 N protein with human plasma positive for IgG antibodies against human coronaviruses NL63, 229E, OC43, and HKU1. There is a cross-reactivity between SARS-CoV positive human plasma and SARS-CoV-2 N protein [20]. The patients in this study have not been infected with SARS-CoV. We also confirmed the specificity of N and S antigens that we used in our study by checking cross-reactivity with serum samples from people infected with other human coronaviruses and human adenovirus, from people vaccinated with influenza virus vaccine and from early collections. There was also a report of serum detection of N specific IgM, IgG and IgA in 135 patients of lower respiratory infection and 150 healthy individuals, all showed no reactivity with SARS-COV-2 N proteins [20].

The clinical symptoms, characteristics, and progression of the 27 non-ICU patients and 11 ICU patients in this study were similar to those of the previously reported COVID-19 patients [10,13,24–26]. Recently, a retrospective study found that the critically ill patient’s fatality rate reached 61.5% within 28 days, and the median time from ICU to death was 7 days [27]. A case of COVID-19 death study found that the CRP increased significantly after the onset of the patient, and lasted for more than 14 days [11]. In our study, CRP also increased significantly in most patients. The increase of S-IgG in non-ICU patients positively correlated with the decrease of CRP, which has not been reported before. Notably, in the third week after symptom onset, N-IgG was significantly higher while S-IgG is significantly lower in ICU patients than in non-ICU patients. Non-ICU patients tend to produce S-IgG antibodies, while ICU patients tend to produce N-IgG antibodies. Interestingly, S-IgG had a parallel or similar dynamic pattern as S-IgM in the first two weeks, but S-IgG continued to increase in the third week while S-IgM in some patients showed plateau or decline in some patients. The similar pattern also occurred in non-ICU patients, but not in ICU patients. This result suggested that the early class switching of IgM to IgG may help predict a better outcome of COVID-19 disease.

It has been recognized that S-specific antibodies can block the binding of S protein to cellular receptor hACE2 that mediates SARS-CoV-2 binding and entry to target cells. There was no evidence that N-specific antibodies can block virus infection. N protein is a suitable candidate for early diagnosis of infection, due to its high immunogenicity and intracellular accumulation before the packaging of the virus [28–30]. A previous study on SARS-CoV infection indicated that IgG response is directed most frequently and predominantly at the N protein (89%), but not S protein (63%) [31]. In this study, we found that most ICU patients had higher N-IgG than S-IgG after the symptom onset, which may be caused by longer and a large amount of virus exposure in the early infections of ICU patients. It is important to note that ICU patients had SARS-CoV-2 nucleic acid positive days of 31.0, whereas non-ICU patients had SARS-CoV-2 nucleic acid positive days of 13. Therefore, a continuous increase of N-IgG may indicate disease progression towards more severe illness. In contrast, S-IgG increased slowly in ICU patients. Research about the contributions of the structural proteins of SARS-CoV to protective immunity indicated that only S protein induced a high titre of SARS-CoV-neutralizing antibodies and protective efficacy in hamsters, but not N protein, matrix M and small envelope E proteins [32]. Homology modelling and structural evidence revealed that SARS-CoV-2 had a similar receptor-binding domain structure to that of SARS-CoV, despite amino acid variation at some key residues [2,3]. In this study, we found that S-IgG in ICU patients was significantly lower than non-ICU patients by 2 weeks after the onset, which may explain the longer hospital stays and nucleic acid positive days in ICU patients. Therefore, monitoring the kinetics of S-IgG should help to predict prognosis.

## Supplementary Material

Supplemental Material
