# Supplementary figures and images for: Kinetics of SARS-CoV-2 specific IgM and IgG responses in COVID-19 patients
Source: Emerg Microbes Infect. 2020 May 13;9(1):940–8. doi: 10.1080/22221751.2020.1762515 (PMC7273175; doi:10.1080/22221751.2020.1762515)

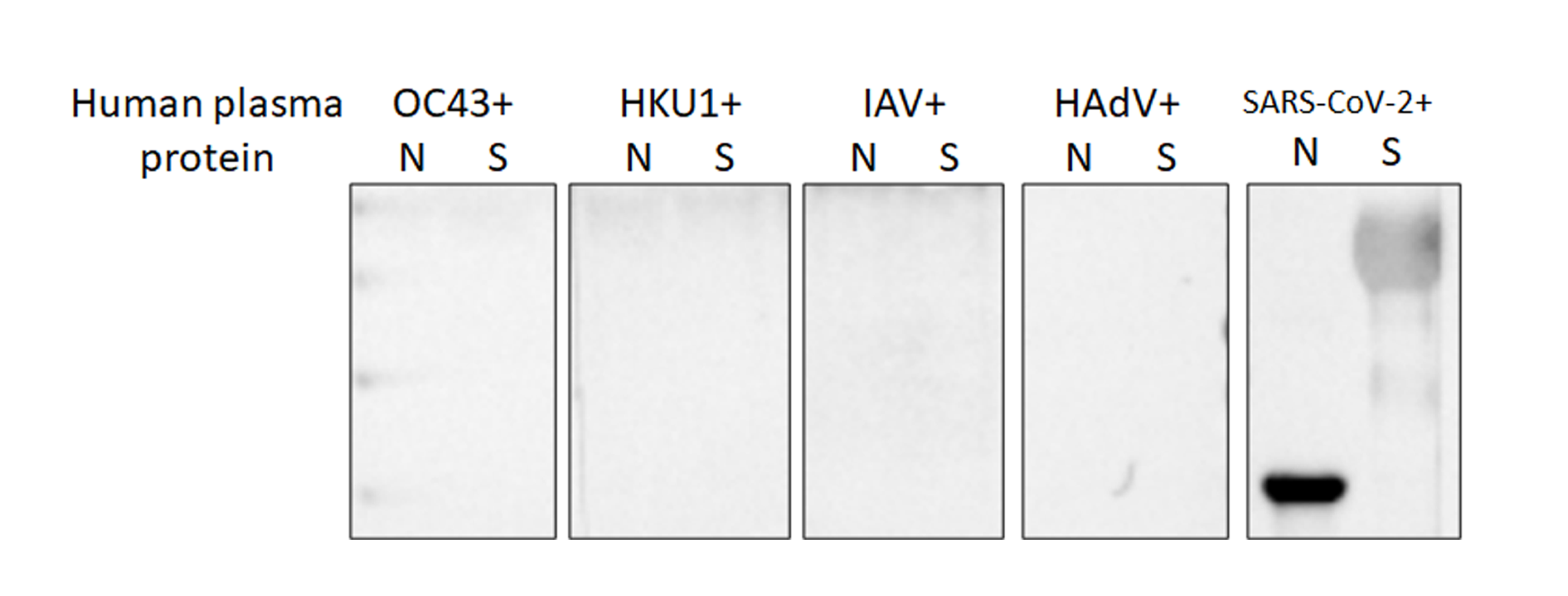

Supplement: Supplemental Material [file TEMI_A_1762515_SM6945.zip › 1762515_supplementary files/supplementary figure1.tif]

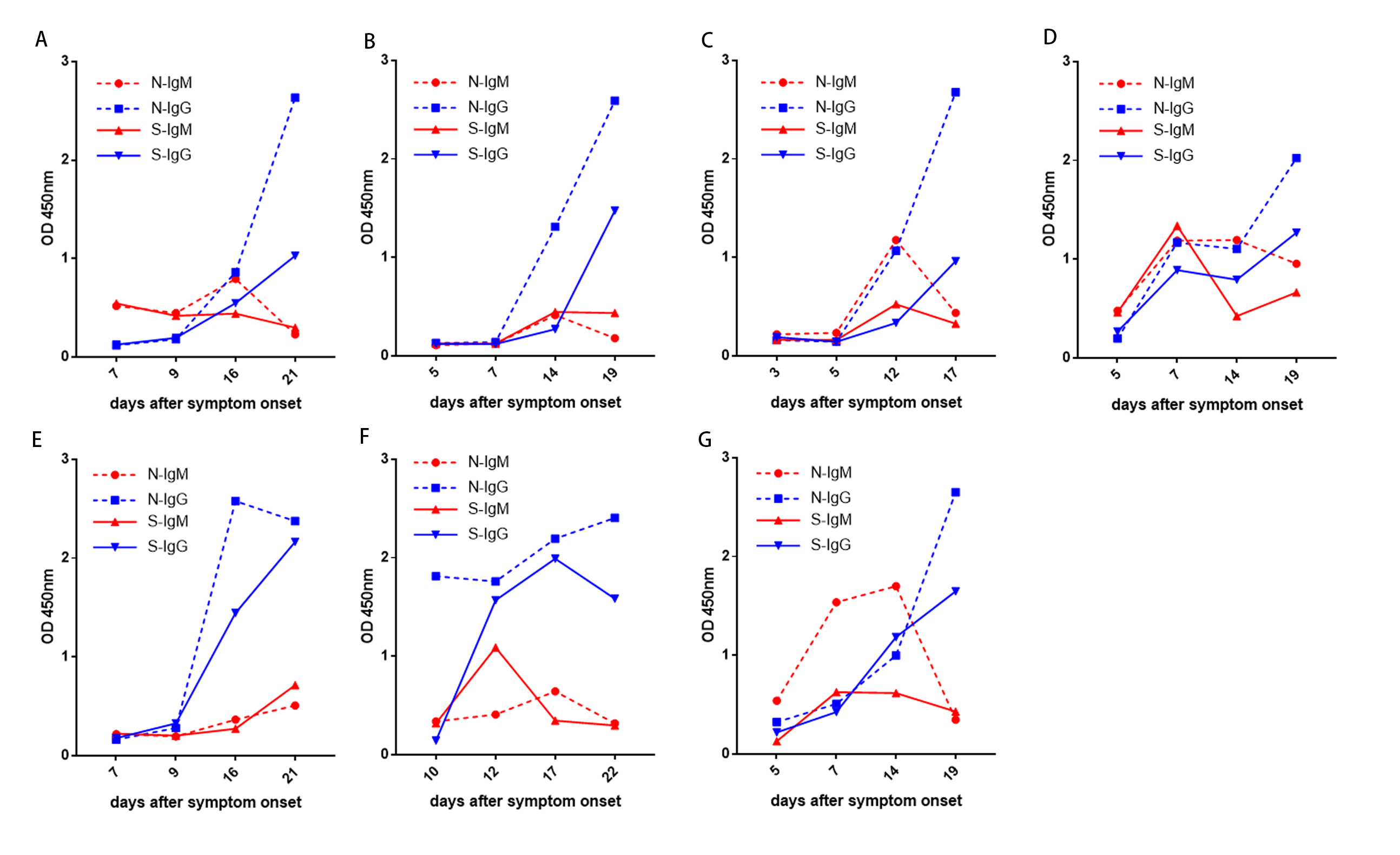

Supplement: Supplemental Material [file TEMI_A_1762515_SM6945.zip › 1762515_supplementary files/supplementary figure2.tif]

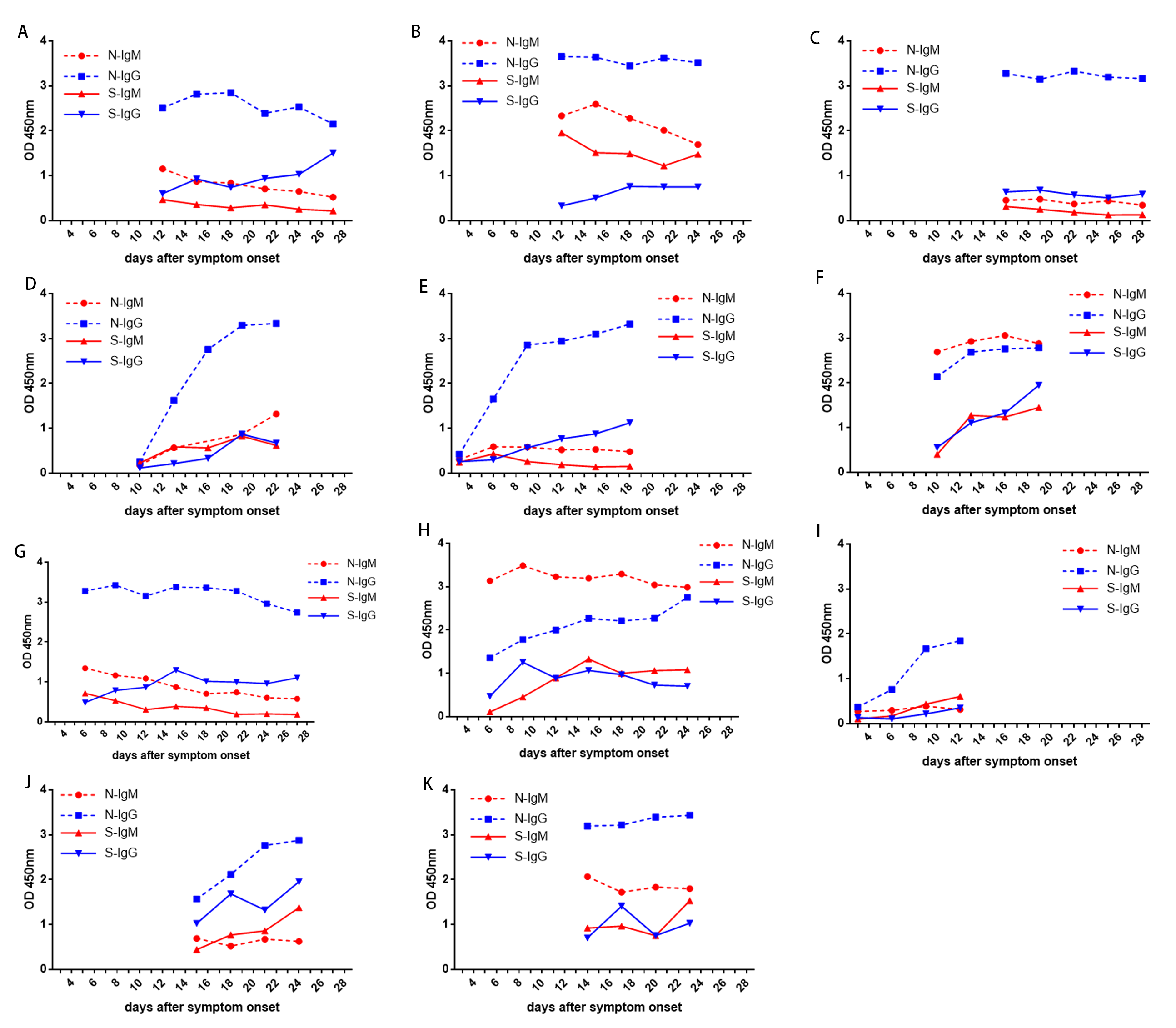

Supplement: Supplemental Material [file TEMI_A_1762515_SM6945.zip › 1762515_supplementary files/supplementary figure3.tif]

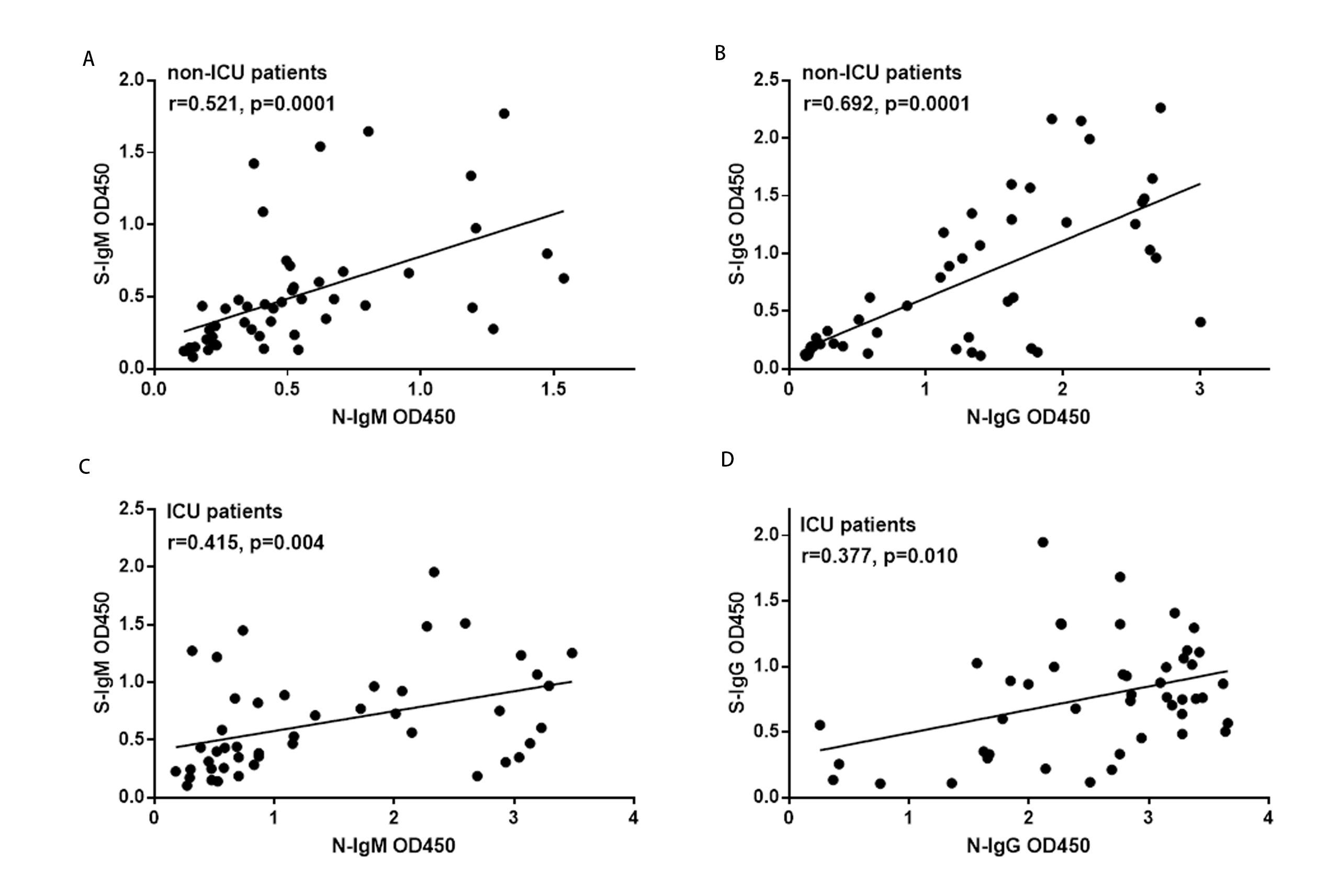

Supplement: Supplemental Material [file TEMI_A_1762515_SM6945.zip › 1762515_supplementary files/supplementary figure4.tif]
